# Supplementary material for: Sorting at embryonic boundaries requires high heterotypic interfacial tension
Source: Nat Commun. 2017 Jul 31;8:157. doi: 10.1038/s41467-017-00146-x (PMC5537356; doi:10.1038/s41467-017-00146-x)
Supplement: Supplementary file 2 — Supplementary Software 1 [file 41467_2017_146_MOESM2_ESM.zip › PottsModel/SrcPottsModel/doc/mvc/class-use/Observer.html]

Uses of Interface mvc.Observer


JavaScript is disabled on your browser.


Skip navigation links


- Overview
- Package
- Class
- Use
- Tree
- Deprecated
- Index
- Help

- Prev
- Next

- Frames
- No Frames

- All Classes

## Uses of Interface mvc.Observer

- Packages that use Observer

  | Package | Description |
  | --- | --- |
  |  |  |
  | --- | --- |
  | engine |  |
  | gui |  |
  | model |  |
  | mvc |  |
- - ### Uses of Observer in engine

    Classes in engine that implement Observer

    | Modifier and Type | Class and Description |
    | --- | --- |
    |  |  |
    | --- | --- |
    | `class` | `PottsLogger` Potts Automaton Logger. |
    | `class` | `StatisticsManager` Manages statistics by initailizing and evaluating the Statistics according to the frequency defined by the user in the simulation settings file. |
  - ### Uses of Observer in gui

    Classes in gui that implement Observer

    | Modifier and Type | Class and Description |
    | --- | --- |
    |  |  |
    | --- | --- |
    | `class` | `PlotPanel` |
    | `class` | `PottsCanvas` Graphical representation of the Potts Model Lattice. |
    | `class` | `PottsFrame` |
    | `class` | `SnapshotManager` Graphical representation of the Potts Model Lattice. |
    | `class` | `StatusBar` |
  - ### Uses of Observer in model

    Classes in model that implement Observer

    | Modifier and Type | Class and Description |
    | --- | --- |
    |  |  |
    | --- | --- |
    | `class` | `Lattice` |
  - ### Uses of Observer in mvc

    Methods in mvc with parameters of type Observer

    | Modifier and Type | Method and Description |
    | --- | --- |
    |  |  |
    | --- | --- |
    | `void` | IObservable.`addObserver(Observer pObserver)` Add an observer to the observable object. |
    | `void` | AObservable.`addObserver(Observer pObserver)` Add an observer to the observable object. |
    | `void` | AObservable.`removeObserver(Observer pObserver)` |

Skip navigation links


- Overview
- Package
- Class
- Use
- Tree
- Deprecated
- Index
- Help

- Prev
- Next

- Frames
- No Frames

- All Classes
